# Association Between Antimicrobial Susceptibility Test Results and Bacteriological Cure of Mild and Moderate Bovine Clinical Mastitis

**DOI:** 10.3390/vetsci13070667

**Published:** 2026-07-09

**Authors:** Breno Luis Nery Garcia, Carlos Eduardo Fidelis, Kristian da Silva Barbosa, Gustavo Freu, Marcos Veiga dos Santos

**Affiliations:** 1 Faculty of Veterinary Medicine, University of Calgary, Calgary, AB T2N 1N4, Canada; brenoluis.garcia@ucalgary.ca; 2 Department of Animal Nutrition and Production, School of Veterinary Medicine and Animal Science, University of São Paulo, Pirassununga 13635-900, São Paulo, Brazil; carlosfidelis@usp.br (C.E.F.); barbosa.kristian@usp.br (K.d.S.B.); gustavo.freu@ourofino.com (G.F.)

**Keywords:** antimicrobial resistance, mastitis, antimicrobial susceptibility testing

## Abstract

Antimicrobial resistance (AMR) is a global concern partly driven by excessive antimicrobial use (AMU) in animal production. Clinical mastitis (CM) is a leading cause of AMU in dairy farms, highlighting its importance from a public-health perspective. This observational study aimed to evaluate the use of antimicrobial susceptibility testing as a criterion for selecting antimicrobials for the treatment of mild and moderate CM in dairy cows. To this end, the association between in vitro antimicrobial susceptibility test results of mastitis-causing pathogens and in vivo bacteriological cure (BC) was assessed. A total of 340 cows with mild or moderate CM from 14 commercial dairy herds in Brazil were enrolled in the study. Isolates from the pretreatment milk samples were subjected to antimicrobial susceptibility testing (AST) using agar disk diffusion. Susceptibility results and cow-level variables were included in a generalized linear mixed model (GLMM) to evaluate their association with BC. No significant association was found between the antimicrobial susceptibility results and BC of the mastitis cases. In conclusion, although in vitro susceptibility testing remains an important tool for AMR surveillance, the findings of this study suggest that its utility in guiding antimicrobial treatment decisions in CM cases is limited.

## Simple Summary

Antimicrobial resistance (AMR) is a growing public health concern, partly driven by the overuse of antimicrobials in livestock. In the dairy industry, responsible antimicrobial use for treating clinical mastitis (CM) can help reduce AMR risk. This study evaluated the association between antimicrobial susceptibility testing results and bacteriological cure (BC) outcomes in mild and moderate CM cases. In total, 340 mild or moderate CM cases were assessed, and no clear overall association was observed between the antimicrobial susceptibility results and BC.

## 1. Introduction

Antimicrobial resistance (AMR) is an emerging global public health issue that requires immediate attention [1]. The increase in AMR has been partially associated with antimicrobial use (AMU) in animal production [2,3]. Restricting AMU in livestock has been associated with a reduction in AMR levels among bacterial isolates from both animals and humans, particularly those directly exposed to food-producing animals [4].

In the dairy industry, although AMU is highly regulated in most developed dairy regions [5], such practices have not been adopted globally, highlighting the ongoing need for antimicrobial stewardship. Antimicrobial therapy remains a primary strategy for treating infectious diseases, with clinical mastitis (CM) treatment and dry cow therapy being the main drivers of AMU [6,7,8]. Although clear evidence of a direct link between AMU for mastitis treatment and AMR in human health remains lacking, increasing public pressure to reduce AMU in livestock, highlighted by the Global Action Plan on AMR established by the World Health Organization [9], underscores the need for measures that promote the rational use of antimicrobials in the treatment of CM in dairy herds.

The adoption of microbiological identification methods to enable selective CM therapy [10,11,12] and the development of methods to assist in the judicious selection of antimicrobials for CM treatment are critical steps in addressing this issue. This is particularly relevant for preventing the use of clinically important antimicrobials commonly used in human medicine in dairy production. These antimicrobials are generally unnecessary for treating mastitis [13], although their use is regulated in some countries [5]. In other countries, such as Brazil, they remain widely used [7], potentially contributing to the emergence and spread of antimicrobial resistance [3].

Continuous monitoring of the antimicrobial susceptibility of mastitis pathogens through AST, such as the agar disk diffusion method, may support judicious AMU on dairy farms. However, previous studies have reported inconsistent or weak associations between AST results and bacteriological cure (BC) under lab conditions [14,15,16]. Nevertheless, most CM cases are still treated empirically without prior susceptibility information [17], indicating limited integration of resistance data into treatment decisions. Incorporating on-farm–suitable AST may improve treatment accuracy and support more rational antimicrobial use in dairy herds.

Based on this context, we hypothesized that the occurrence of AMR, particularly multidrug resistance in mastitis-causing pathogens identified through AST, reduces the likelihood of BC in mild and moderate CM cases treated with antimicrobials. Therefore, this study aimed to evaluate the use of antimicrobial susceptibility testing as a criterion for selecting antimicrobials for the treatment of mild and moderate CM in dairy cows, with the broader goal of contributing to improved antimicrobial stewardship in dairy herds.

## 2. Materials and Methods

### 2.1. Selection of Herds and Cows

Five dairy herds averaging approximately 1210 lactating cows and producing 31.2 kg/cow/day were selected for the present study. The herds, located in the states of São Paulo (*n* = 4) and Minas Gerais (*n* = 1), Brazil, routinely used on-farm culture (OFC) for selective treatment protocols for CM cases and were chosen based on convenience sampling (proximity to the Milk Quality Research Laboratory at SVMAS-USP and willingness to participate in the study). Additionally, 59 isolates (17.3%; 59/340 of all isolates included in the study) from pre-collected milk samples of CM cases obtained in a previous study were included. These samples, collected between 2019 and 2021 from nine dairy farms (two in São Paulo and seven in Minas Gerais, Brazil), followed the same diagnostic and inclusion procedures as the present study. The combined dataset therefore includes isolates originating from 14 dairy herds. All isolates were stored at −80 °C in the mastitis pathogen collection of the Milk Quality Research 80 °C at SVMAS-USP until analysis.

### 2.2. Clinical Mastitis Diagnosis and Milk Sample Collection

Clinical mastitis was defined as the presence of abnormalities in milk and/or the mammary quarter according to the following severity score system: (a) mild, score 1: alterations in milk appearance (e.g., presence of clots or flakes in the strip cup test, watery consistency, or abnormal coloration); (b) moderate, score 2: pain, heat, and localized swelling of the affected quarter with or without changes in milk appearance; and (c) severe, score 3: symptoms of scores 1 and 2, plus systemic symptoms (e.g., fever and loss of appetite). Only cows with scores of 1 and 2 were included in this study.

Cows were excluded from the study for the following reasons: (a) presence of severe systemic clinical signs requiring treatments other than intramammary antibiotic therapy; (b) concurrent diseases (e.g., hoof problems, metritis, pneumonia, and retained placenta); (c) cows that had received any antimicrobial treatment (systemic or intramammary) within 15 days prior to the study; and (d) cows with more than three CM cases in the same quarter during the same lactation.

Clinical mastitis cases were diagnosed visually by trained farm employees. Milk samples from the affected mammary quarters were collected prior to treatment, in accordance with the National Mastitis Council (NMC) guidelines [18]. Additionally, the following information was recorded for each CM case: (a) CM severity score, (b) affected mammary quarter, (c) treatment protocol administered, (d) treatment duration, (e) days in milk (DIM), (f) history of CM during lactation, (g) parity, (h) average milk production, (i) cow breed, and (j) clinical cure.

**Figure 1:**
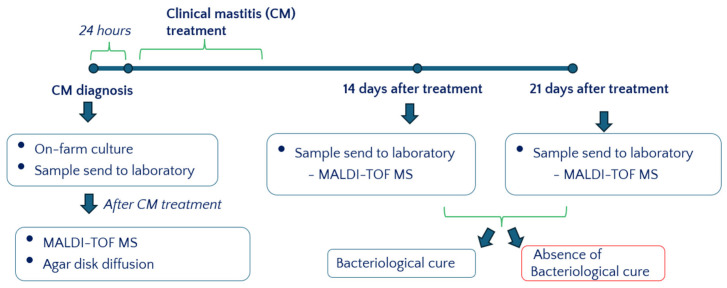
Schematic representation of milk sample collection and laboratory analysis schedule according to the study protocol.

After aseptic sample collection, which was part of the herd’s routine mastitis diagnosis, milk samples from cows with CM were inoculated directly onto OFC chromogenic culture media by farm personnel, as described by Granja et al. [12]. Briefly, bacterial growth was identified using Smart Color 2 chromogenic triplicate plates (OnFarm^®^, Piracicaba, Brazil), with milk inoculation carried out using a sterile swab. The plates were incubated at 37 °C in an incubator located on each farm (SmartLab, OnFarm^®^, Piracicaba, Brazil), and microbial growth was observed after 24 h of incubation. The OFC inoculation was performed immediately after milk collection, whereas milk samples from the mammary quarters were stored at −20 °C and subsequently sent to the microbiology laboratory for further analysis. Treatment protocols were initiated immediately after microbiological identification (i.e., approximately 24 h after diagnosis; Figure 1).

### 2.3. Clinical and Bacteriological Cure of Clinical Mastitis

Clinical cure of CM was assessed and recorded by trained personnel from the participating farms. Clinical cure was defined as the complete absence of clinical signs of CM following the completion of the treatment protocol and sustained absence of symptoms for up to 21 days after treatment initiation. Clinical cases occurring in the same mammary quarter after this interval were classified as new intramammary infections (NIMIs) [19], whereas those occurring within this interval were considered treatment failures. Recurrent cases were defined as more than one but fewer than three CM episodes in the same mammary quarter within the same lactation, with at least 21 days between episodes. Cows with more than three CM cases in the same mammary quarter within the same lactation were classified as having chronic CM [19] and excluded from the study.

For the evaluation of BC, milk samples were collected at three time points: pretreatment (day 0, D0), and at 14 ± 3 days (D14), and 21 ± 3 days (D21) post-treatment. Pretreatment samples were collected for OFC and frozen for shipment to the laboratory along with post-treatment samples. Bacteriological cure was defined as the isolation of a pure microorganism culture before treatment (D0) and the absence of isolation of the same microorganism in all post-treatment samples (D14 and D21). If a different species was isolated in the post-treatment samples compared to the pretreatment samples, the case was considered bacteriologically cured with a NIMI [20].

### 2.4. Microbiological Identification of the Mastitis-Causing Agents and Antimicrobial Susceptibility Testing

Milk samples from CM cases were collected at pretreatment (D0, using the same samples collected for OFC in the participant herds) and post-treatment (D14 and D21), frozen, and sent to the laboratory for microbiological identification. Briefly, milk samples were inoculated onto blood agar and incubated at 37 °C for 24 to 48 h, according to the National Mastitis Council (NMC, [18]) guidelines. Bacterial isolates were identified using matrix-assisted laser desorption/ionization time-of-flight mass spectrometry (MALDI-TOF MS; Bruker Daltonics Inc., Bremen, Germany) according to Barcelos et al. [21]. Species-level identification was considered when the sample score was >2, genus-level identification when the score was >1.7 and <2, and unreliable identification when the score was <1.7. A cutoff score of 1.7 was established for the microbiological identification of isolates in this study [22], with genus-level microbiological identification (1.7–2) being accepted only for minor pathogens [23].

### 2.5. Antimicrobial Susceptibility Testing with Agar Disk Diffusion

Clinical mastitis isolates underwent in vitro antimicrobial susceptibility testing using agar disk diffusion, following the recommendations of the Clinical and Laboratory Standards Institute [24].

Briefly, following isolation, one to three colonies grown on blood agar plates were inoculated into 5 mL of sterile saline solution, mixed by vortexing, and adjusted to 0.5 McFarland (approximately 10^8^ CFU/mL) using a turbidimeter (Uniscience, Osasco, São Paulo, Brazil). This standardized suspension was uniformly inoculated onto two Mueller–Hinton agar plates per isolate using a sterile swab. Antimicrobial disks were placed on the plates, with 12 disks per isolate (six per plate).

The panel of antimicrobials reflected the most commonly used intramammary products in Brazil [7,25,26], including enrofloxacin (5 µg), kanamycin (30 µg), penicillin–novobiocin (10 µg), oxacillin (30 µg), ceftiofur (30 µg), gentamicin (10 µg), neomycin (30 µg), ampicillin (10 µg), clindamycin (2 µg), cephalexin (30 µg), amoxicillin–clavulanate (30 µg), and tetracycline (30 µg).

After incubation at 37 °C for 24 h, the Scan 1200 Interscience 8.8 software of the automated colony counter (Scan 1200, Interscience, Paris, France) was used for inhibition zone measurement. Antimicrobial susceptibility testing was performed following the recommendations of the Clinical and Laboratory Standards Institute (CLSI, 2025) and the European Committee on Antimicrobial Susceptibility Testing (EUCAST, 2025). Standard reference strains (*Staphylococcus aureus* ATCC 29213 and *Escherichia coli* ATCC 25922) were used for quality assurance. Assays yielding out-of-range control results were repeated in their entirety to verify accuracy.

### 2.6. Antimicrobial Susceptibility Test Inhibition Zone Diameter Interpretation

Inhibition zone diameters measured on Mueller–Hinton agar plates were interpreted according to the Clinical and Laboratory Standards Institute [24,27] and EUCAST [28] breakpoints. For analytical purposes, isolates classified as intermediate were grouped with resistant isolates and considered non-susceptible, as the intermediate category represents uncertain clinical efficacy under field conditions. This approach is widely adopted in antimicrobial resistance surveillance and epidemiological studies because it provides a conservative classification of susceptibility and facilitates binary statistical analyses.

Interpretation of inhibition zones followed CLSI VET01S and EUCAST Version 15.0 guidelines where species-specific veterinary breakpoints existed. When veterinary breakpoints were unavailable, human clinical breakpoints were applied [27]. Where antimicrobial-species combinations lacked defined criteria, the breakpoints of a structurally related agent within the same drug class were substituted, a pragmatic approach necessitated by the scarcity of validated mastitis-specific interpretive standards [14]. A detailed overview of all antimicrobial–pathogen combinations and the corresponding interpretive criteria used for breakpoint selection is provided in Table 1.

Isolates lacking species-specific interpretive guidelines were classified into one of four phenotypic categories: *Staphylococcus* spp., *Streptococcus* spp., and *Enterococcus* spp. for Gram-positive cocci, and *Enterobacterales* for Gram-negative rods. Isolates phylogenetically related to *Streptococcus* spp. (specifically those belonging to the *Lactococcus* and *Aerococcus* genera) were grouped as “Strep-like bacteria” for analytical purposes. Susceptibility results for these isolates were interpreted using *Streptococcus* breakpoints based on their phylogenetic relationship within the order *Lactobacillales* and the absence of validated genus-specific veterinary interpretive criteria [29,30].

### 2.7. Sample Size Calculation

To detect a 15% difference in microbiological cure between treatments, a sample size of 260 CM cases was determined (Sealed Envelope Ltd. 2012; Power calculator for binary outcome superiority trials. Available from: https://www.sealedenvelope.com/power/binary-superiority/ [Accessed Mon 9 June 2025]), assuming a type I error of 0.05 and a type II error of 0.2. To account for an estimated 15% loss of cases during the study, the final experimental sample size was set to 299 CM cases.

### 2.8. Statistical Analysis

The association between the results of antimicrobial susceptibility tests and CM BC was assessed using a generalized linear mixed model (GLMM). The model was fitted with a binomial distribution and a logit link function with the glmer function from the lme4 package [31] in the R studio software (version 4.1.3). Data processing and DataFrame handling were performed in Python (version 3) using the pandas library [32], with the binary outcome variable defined as bacteriological cure of CM (1 = cured, 0 = not cured). All independent variables were initially screened using univariate logistic regression models. Variables with a *p* ≤ 0.30 in the univariable analysis and those considered biologically relevant were selected for inclusion in the multivariable model. The coefficients of the primary variables in the full model were compared with their estimates in the reduced models, excluding one potential confounding variable at a time. If the exclusion of a variable resulted in a variation of more than 25%, the variable was retained in the final model to account for its influence.

The following independent variables were included in the final model: (a) CM severity score (mild or moderate); (b) parity; (c) mastitis-causing pathogen, grouped as: *Staphylococcus* spp., *Streptococcus* spp., Strep-like bacteria, and Gram-negative bacteria; (d) treatment protocol (five categories representing the most commonly used protocols, with protocols having <20 observations grouped as “other treatment protocols”), (e) multidrug resistance status (yes or no) and (f) number of antimicrobial resistances observed in the in vitro susceptibility test. Herd was included as a random effect to account for the clustering of observations within farms.

The distributions of all independent variables were checked for balance prior to the modeling. Multicollinearity among categorical predictors was assessed using the variance inflation factor (VIF), and no evidence of high multicollinearity (VIF > 5) was observed among the model explanatory variables. Additionally, a Pearson correlation matrix was performed to assess multicollinearity among continuous variables, and no high levels of collinearity (>0.8 or <−0.8) were observed. Model fit was evaluated using the Akaike information criterion (AIC), and the model’s convergence was assessed using the default Nelder–Mead optimizer.

The GLMM is described as follows:

logit (pi) = β_0_ + β_1_ × (Severity) + β_2_ × (Parity) + β_3_ × (Pathogen Group) + β_4_ × (Treatment protocol) + β_5_ × (MDR Status) + β_6_ × (Number of resistances) + Herd (random) + RE

In these equations, logit (pi) is the log-odds of BC of CM; β0 is the intercept; β_1_ is the fixed effect for the categorical variable CM severity score; β_2_ represents the fixed effect for parity, included as a continuous covariate; β_3_ corresponds to the fixed effects of the categorical mastitis-causing pathogen groups (*Staphylococcus* spp., *Streptococcus* spp., Strep-like bacteria, and Gram-negative bacteria); β_4_ is the fixed effect of the categorical treatment protocol variable; β_5_ is the fixed effect of multidrug resistance status of the bacterial isolate (yes or no); and β_6_ is the fixed effect of the continuous variable of the number of antimicrobial resistances observed in the in vitro susceptibility test. The herd was included as a random effect, and RE denoted the residual error.

Additionally, at the pathogen level, the association between in vitro antimicrobial susceptibility test results and BC in vivo was evaluated using a logistic regression model. A multivariable logistic regression model was fitted using the maximum likelihood estimation method with the stats models package [33] in Python (version 3) and the model fit was evaluated using the pseudo-R^2^, likelihood ratio, and assessment of individual coefficient significance. For each pathogen group (*Staphylococcus* spp., *Streptococcus* spp., Strep-like bacteria, and Gram-negative bacteria), the binary outcome of susceptibility to each antimicrobial agent tested (1 = resistant, 0 = sensitive) was treated as an independent variable. BC in vivo (1 = cured, 0 = not cured) was treated as the dependent variable according to the following model:

logit (pi) = β_0_ + β_1_ × (ENF) + β_2_ × (KAN) + β_3_ × (PEN) + β_4_ × (OXA) + β_5_ × (CFF) + β_6_ × (GEN) + β_7_ × (NEO) + β_8_ × (AMP) + β_9_ × (CLI) + β_10_ × (CEF) + β_11_ × (AMC) + β_12_ × (TET)

In this equation, logit (pi) represents the logistic function of BC; β_0_ is the intercept; and β_1_ to β_12_ represent the regression coefficients corresponding to the antimicrobial susceptibility status to the respective antimicrobials evaluated: β_1_ = enrofloxacin (5 µg), β_2_ = kanamycin (30 µg), β_3_ = penicillin-novobiocin (10 µg), β_4_ = oxacillin (30 µg), β_5_ = ceftiofur (30 µg), β_6_ = gentamicin (10 µg), β_7_ = neomycin (30 µg), β_8_ = ampicillin (10 µg), β_9_ = clindamycin (2 µg), β_10_ = cephalexin (30 µg), β_11_ = amoxicillin + clavulanate (30 µg), and β_12_ = tetracycline (1 µg).

Because not all evaluated pathogens have established clinical breakpoints for these antimicrobials, the model was adapted for each pathogen group, excluding variables corresponding to antimicrobials without available interpretative guidelines for zone diameters.

## 3. Results

### 3.1. Descriptive Results and Clinical Mastitis Cases Characterization

From 2019 to 2023, 454 CM cases from 14 dairy herds were included in this study. Of these, 23.3% (114/454) were excluded for the following reasons: (a) no bacterial growth in the pretreatment milk sample (*n* = 44); (b) failure in sample/cow’s data collection or submission (*n* = 40); (c) contamination of the pretreatment milk sample (*n* = 22); (d) early dry period (*n* = 3); (e) culling of the cow or permanent dry off of the mammary quarter (*n* = 4) and (f) cow not being treated for the CM case (*n* = 1).

Of the remaining 340 CM cases, 77.9% (265/340) were classified as mild CM, and 22.1% (75/340) were classified as moderate CM (Table 2). Clinical cure was reported in 85.3% (290/340) of the CM cases, BC was observed in 72.9% (248/340) of the cases, and new intramammary infections were observed in 26.7% (91/340) of the cases. Mastitis-causing pathogen/group level BC is presented in Table 3.

These CM cases were reported in 276 cows, primarily Holsteins (69.7%), with an average daily milk yield of 31.4 L/day. Regarding parity, 27.6% (94/340) of the cows were primiparous, 27.0% (92/340) were in their second lactation, and 45.4% (154/340) were multiparous cows in their third or higher lactation. In 33.4% (114/340) of the cases, CM occurred in early lactation (0–100 DIM); in 32.5% (110/340) of cases, CM occurred during mid-lactation (100–200 DIM); and in 34.1% (116/340), CM occurred in late lactation (after 200 DIM). Clinical mastitis was more frequent in the front mammary quarters (65.3%; 222/340) than in the rear mammary quarters (34.7%; 118/340).

Treatment protocols showed considerable variability (Table 3), with the most frequently used protocol being amoxicillin + clavulanate (28.2%; 96/340) followed by cephalexin + kanamycin (25.3%; 86/340) usage. Due to the large number of protocols with low occurrence, treatments used in fewer than 20 CM cases were grouped into a single category labeled “Other treatments,” comprising all remaining antimicrobial combinations used (Table 2).

A total of 46 bacterial species of mastitis-causing pathogens were isolated from 340 CM samples. The most frequently isolated bacterial genus was *Staphylococcus* spp., which accounted for 34.1% (116/340), followed by *Streptococcus* spp. (30.0% (102/340). The most frequently isolated bacterial species were *Staphylococcus chromogenes* (18.5%, 63/340), followed by *Streptococcus uberis* (14.1%; 48/340) and *Escherichia coli* (12.1%; 41/340; Table 4).

Of the 340 isolates, 148 isolates (43.5%) were susceptible to all antimicrobials, while 192 (56.5%) were resistant to at least one antimicrobial test (Table 5). Multidrug resistance, defined as simultaneous resistance to three or more antimicrobial classes [34], was observed in 44 isolates (12.9%), with an average of 1.3 resistances per isolate.

### 3.2. Generalized Linear Mixed Model and Linear Regression Model Results

A total of 275 CM cases were assessed for BC using a GLMM, considering cow-level and pathogen-level data as independent variables. Of the 340 CM cases, 7% (24/340) were excluded from the analysis due to missing data for the independent variables included in the final model. Additionally, 41 CM cases (12%; 41/340) caused by *E. coli* were excluded from the analysis because of the high rate of spontaneous cure reported for this pathogen [35]. As the objective of the analysis was to evaluate the association between antimicrobial susceptibility status and BC, inclusion of *E. coli* cases could have confounded this relationship. Although these cases were treated with antimicrobials, treatment is generally not recommended for mild and moderate *E. coli* CM cases. Therefore, their exclusion was intended to reduce potential bias in the estimation of treatment-related effects on BC.

The model successfully converged, with an AIC value of 333.8 and no evidence of standardization issues in the residues. Among the explanatory variables, only the treatment protocol category had a statistically significant effect on the binary outcome of bacteriological cure in CM cases. Specifically, the protocols tetracycline + neomycin + bacitracin + sulfadoxine + trimethoprim (*p* = 0.038) and amoxicillin + clavulanate + tylosin (*p* = 0.047) were associated with a higher odds of cure. Neither multidrug resistance status (*p* = 0.087) nor the number of antimicrobial resistances in the in vitro antimicrobial susceptibility test (*p* = 0.70) significantly impacted the outcome (Table 6).

Separate logistic regression models were attempted for each of the four evaluated pathogen groups regarding the pathogen-level association between the in vitro antimicrobial susceptibility test and the likelihood of BC. However, for the groups *Streptococcus* spp., Strep-like bacteria, and Gram-negative bacteria, the models failed to converge, likely because of the limited sample size and unbalanced distribution of susceptibility to the antimicrobials tested. The only pathogen group for which the model successfully converged was *Staphylococcus* spp., with 116 CM cases assessed (Table 7).

**Table 7:** Results of the logistic regression model for the association between in vitro antimicrobial susceptibility test results and in vivo bacteriological cure of clinical mastitis cases caused by *Staphylococcus* spp. (*n* = 116).

Independent Variable	Estimate (β)	SE ^1^	*p*-Value	OR ^2^	95% CI ^3^
Intercept	1.013	0.247	0		(1.7 to 4.47)
Penicillin-novobiocin (10 µg)	0.673	1.267	0.595	1.96	(0.16 to 23.45)
Ampicillin (10 µg)	−1.302	1.12	0.245	0.27	(0.03 to 2.44)
Oxacillin (30 µg)	−1.392	0.703	0.048 *	0.25	(0.06 to 0.99)
Ceftiofur (30 µg)	1.491	1.744	0.393	4.44	(0.15 to 135.33)
Kanamycin (30 µg)	−0.258	1.638	0.875	0.77	(0.03 to 19.09)
Enrofloxacin (5 µg)	0.043	0.965	0.964	1.04	(0.15 to 7.78)
Clindamycin (2 µg)	−0.766	0.653	0.241	0.47	(0.12 to 1.75)
Tetracycline (1 µg)	1.065	0.806	0.186	2.9	(0.45 to 18.57)

^1^ SE: Standard error; ^2^ OR: odds ratio; ^3^ CI: confidence interval. * Statistically significant at the 95% confidence level.

Although the overall model for *Staphylococcus* spp. was not significant (likelihood ratio test: *p* = 0.370; pseudo R^2^ = 0.13), indicating that the susceptibility profile explained only 13% of the variability in the BC, the susceptibility result for oxacillin (30 µg) was significantly associated with BC (*p* = 0.048). *Staphylococcus* spp. isolates resistant to oxacillin had an estimated regression coefficient of −1.39 and an odds ratio of 0.249, suggesting a potentially reduced odds of approximately 75% for BC. None of the other antimicrobials were significantly associated with BC.

## 4. Discussion

The present study evaluated the potential of antimicrobial susceptibility testing as a supportive tool for antimicrobial therapy decision-making in CM. No association was observed between the in vitro antimicrobial susceptibility status of pathogens causing CM and BC, although specifically for *Staphylococcus* spp., oxacillin resistance increased the odds of treatment failure. In this trial, CM treatment protocols were not standardized, which limited the ability to directly correlate in vitro susceptibility test results with the same antimicrobial protocol’s in vivo clinical efficacy. However, the primary objective of this study was not to assess the therapeutic response to specific drugs, which has already been explored in several studies [36,37,38,39], but rather to determine the impact of phenotypic antimicrobial susceptibility status on the likelihood of BC in mastitis-causing pathogens.

The GLMM demonstrated overall significance, denoted by convergence and model fit (AIC = 333.8), indicating that the combined set of independent variables collectively influenced the BC. Among the independent variables, only the treatment protocols were significantly associated with BC. Combined antimicrobial treatment protocols based on tetracycline + neomycin + bacitracin + sulfadoxine + trimethoprim (*p* = 0.038) and amoxicillin + clavulanate + tylosin (*p* = 0.047) were associated with higher BC odds, with odds ratios of 38.9 (95% CI: 1.79–845.99) and 21.0 (95% CI: 1.43–308.38), respectively. However, given the large number of treatment categories and the resulting imbalance in group sizes, these associations may be influenced by the small number of observations per treatment category (*n* = 28 and *n* = 31, respectively), which probably contributed to the wide confidence intervals observed. Notably, both antimicrobial treatment protocols combined different antimicrobial formulations and routes of administration (intramammary and parenteral). These treatment practices suggest the off-label use of antimicrobials, which is generally not recommended, especially in the context of rational antimicrobial use, except in cases of severe mastitis [40], which were not included in this study.

Regarding the antimicrobial susceptibility of the mastitis-causing pathogens, none of the evaluated variables reached statistical significance (*p* > 0.05) when assessed individually. Neither multidrug resistance status nor the number of antimicrobial resistances showed evidence of impacting the likelihood of BC in the evaluated mild and moderate CM cases. Consistent with previous studies [14,15], our findings indicate that in vitro antimicrobial susceptibility assays, such as MIC and agar disk diffusion, are not reliable predictors of treatment outcomes in CM or subclinical mastitis [16]. Similar results were reported by Apparao et al. [15], who found no association between antimicrobial susceptibility determined by broth microdilution and treatment outcomes in CM cases caused by Gram-positive pathogens treated with cephapirin sodium. Likewise, Hoe and Ruegg [14] and Apparao et al. [16] reported weak relationships between in vitro susceptibility results and bacteriological cure in CM and subclinical mastitis cases treated with pirlimycin. Collectively, these findings suggest that mastitis treatment outcomes are influenced by factors beyond phenotypic antimicrobial resistance alone. This poor correlation may be partially attributed to the limitations in the standardization of interpretive breakpoints for mastitis-causing pathogens, which often require the use of nonspecific guidelines or adaptations from different sources of pathogens, as well as the grouping of species together for interpretation [30]. Moreover, certain intrinsic virulence factors of specific species (e.g., *Staphylococcus aureus*), such as biofilm formation and intracellular invasion, can hinder antimicrobial efficacy, leading to treatment failure despite in vitro susceptibility [41,42].

As aforementioned, the wide diversity of treatment protocols administered for CM cases in this study, along with the use of inappropriate or off-label treatments that did not account for pathogen-specific intrinsic resistance, represents a significant limitation of this study. This variability hinders the ability to accurately predict treatment outcomes, as treatment failure may result from the use of ineffective antimicrobials or drugs without labeled indications or expected efficacy against the pathogens involved. For instance, *Escherichia coli* isolates (*n* = 41) were excluded from the analysis because their high rate of spontaneous cure could have acted as a confounder in the interpretation of BC in treated mild and moderate-CM cases. This exclusion reduced the final sample size to below the number estimated in the power calculation and may have limited the statistical power of the GLMM, increasing the risk of type II errors. This may have hindered the model’s predictive capacity, thereby limiting the interpretation of the results. However, when *Escherichia coli* CM cases were included in the analysis, no significant differences were observed in the effects of the explanatory variables on BC. Additionally, in the pathogen-level analyses, sparse data for some pathogen groups contributed to model instability, wide confidence intervals, and the inability to achieve convergence in several pathogen-specific models. Therefore, the absence of significant associations should be interpreted with caution, as some effects may have remained undetected due to limited statistical power.

Although oxacillin resistance was associated with reduced odds of BC in *Staphylococcus* spp., the overall predictive capacity of the antimicrobial susceptibility profile in this model was limited. The overall model was not statistically significant in the likelihood ratio test (*p* = 0.370), and only 13% of the variability could be explained by susceptibility test results (pseudo-R^2^ = 0.13). Oxacillin resistance is commonly linked to methicillin-resistant *Staphylococcus aureus* (MRSA), according to CLSI and EUCAST guidelines. Although only 12 out of 116 *Staphylococcus* spp. isolates were identified as *S. aureus*, most of the isolates were non-aureus *Staphylococcus* (NAS), with *S. chromogenes* representing 18.5% of the total isolates. Among the *S. aureus* isolates, only two (2/12) were oxacillin-resistant, while most oxacillin-resistant isolates were identified as *S. sciuri*, with nine resistant isolates (9/15). Given the non-significant overall model and the absence of molecular confirmation of *mecA/mecC* genes, these results should be interpreted strictly as phenotypic resistance patterns with caution. Nevertheless, these findings suggest an increase in methicillin resistance among certain NAS species causing mastitis, a trend supported by previous studies evaluating AMR in NAS [43,44].

Overall, the results of this study reflect the multifactorial complexity involved in achieving BC for CM and suggest that, in the absence of reliable antimicrobial susceptibility data to guide antimicrobial selection, it becomes even more critical, within the context of judicious AMU, to define the cases of CM that are likely to benefit from and have a higher probability of responding to antimicrobial therapy. In this regard, cow-level factors and the characteristics of the causative pathogen must be considered in decision-making [40].

Importantly, mastitis-causing pathogens differ significantly in virulence and may harbor intrinsic resistance to certain antimicrobials (e.g., intrinsic resistance to several β-lactams in Gram-negative pathogens), which requires targeted and appropriate therapeutic protocols to treat the infection. Notably, microbiological identification of the mastitis-causing pathogen alone can reduce AMU by up to 50% without negatively impacting BC [8] and can further support treatment decisions based on the characteristics of the pathogen species or groups. Although, in our study, in vitro antimicrobial susceptibility results were not associated with BC, suggesting they should be interpreted with caution for CM treatment decisions, these assays remain essential tools for AMR surveillance and monitoring resistance trends in mastitis pathogens [17,45,46].

## 5. Conclusions

This study assessed the potential of antimicrobial susceptibility tests as a tool to support antimicrobial treatment decisions in CM. No association was observed between the antimicrobial susceptibility status of the mastitis-causing pathogens and BC. Although an association between oxacillin resistance and the likelihood of BC in CM caused by *Staphylococcus* spp. was identified, the overall predictive performance of the model was limited. These findings are consistent with previous studies and highlight the complex and multifactorial nature of CM treatment outcomes. Given the substantial heterogeneity in treatment protocols and the lack of treatment standardization among herds, the relationship between in vitro susceptibility results and BC should be interpreted with caution. While antimicrobial susceptibility testing remains essential for AMR surveillance and monitoring resistance trends, its value for predicting BC under field conditions appears limited in the context of the present study. Nevertheless, they underscore the importance of on-farm pathogen identification and antimicrobial stewardship for the selective therapy of CM.

## Figures and Tables

**Table 1:** Adopted genus/group-level clinical breakpoints for interpretation of antimicrobial susceptibility results in mastitis-causing pathogens without species-level clinical breakpoints.

Isolate Group	Antimicrobial	Class	Specie	Source
*Staphylococcus* spp.	Enrofloxacin	Fluoroquinolone	Dog	CLSI supplement VET01S
	Kanamycin	Aminoglycoside	Human	CLSI—M100
	Penicillin	Beta-lactam	Cow	CLSI supplement VET01S
	Oxacillin	Beta-lactam	Human	CLSI—M100
	Ceftiofur	Beta-lactam	Cow	CLSI supplement VET01S
	Gentamicin	Aminoglycoside	Human	CLSI—M100
	Neomycin	Aminoglycoside	Human	CLSI—M100
	Ampicillin	Beta-lactam	Human	EUCAST Version 15.0
	Clindamycin	Lincosamide	Dog	CLSI supplement VET01S
	Amikacin	Aminoglycoside	Human	EUCAST Version 15.0
	Tetracycline	Tetracycline	Dog	CLSI supplement VET01S
*Streptococcus* spp. and Strep-like bacteria	Enrofloxacin	Fluoroquinolone	Dog	CLSI supplement VET01S
	Penicillin	Beta-lactam	Cow	CLSI supplement VET01S
	Oxacillin	Beta-lactam	Cow	CLSI supplement VET01S
	Ceftiofur	Beta-lactam	Cow	CLSI supplement VET01S
	Ampicillin	Beta-lactam	Human	CLSI—M100
	Clindamycin	Lincosamide	Human	CLSI—M100
	Cephalexin	Beta-lactam	Human	CLSI—M100
	Amikacin	Aminoglycoside	Human	CLSI—M100
	Tetracycline	Tetracycline	Human	CLSI—M100
*Enterococcus* spp.	Enrofloxacin	Fluoroquinolone	Human	EUCAST Version 15.0
	Penicillin	Beta-lactam	Human	CLSI—M100
	Ampicillin	Beta-lactam	Human	CLSI—M100
	Clindamycin	Lincosamide	Human	EUCAST Version 15.0
	Tetracycline	Tetracycline	Human	CLSI—M100
Enterobacterales	Enrofloxacin	Fluoroquinolone	Dog	CLSI supplement VET01S
	Kanamycin	Aminoglycoside	Human	CLSI—M100
	Ceftiofur	Beta-lactam	Cow	CLSI supplement VET01S
	Gentamicin	Aminoglycoside	Dog	CLSI supplement VET01S
	Neomycin	Aminoglycoside	Dog	CLSI supplement VET01S
	Ampicillin	Beta-lactam	Human	CLSI—M100
	Cephalexin	Beta-lactam	Human	CLSI—M100
	Amikacin	Aminoglycoside	Human	CLSI—M100
	Tetracycline	Tetracycline	Human	CLSI—M100

**Table 2:** Descriptive results of clinical, bacteriological, and cow-level variables in 340 clinical mastitis cases.

Variable		Number of Observations	%
Clinical cure		290	85.3
Overall bacteriological cure		248	72.9
Bacteriological culture by major pathogen/groups			
	*Staphylococcus aureus*	7	58.3
	Non-*aureus* staphylococci	73	70.2
	*Streptococcus uberis*	31	64.6
	*Streptococcus dysgalactiae*	34	77.3
	*Enterococcus* spp.	11	61.1
	*Escherichia coli*	37	90.2
	*Klebsiella* spp.	15	75.0
NIMI ^1^		91	26.6
Clinical mastitis score			
	Mild	265	77.9
	Moderate	75	22.1
Recurrence ^2^		153	44.9
Breed			
	Holstein	237	69.7
	Crossbreed	103	30.3
Parity			
	1	94	27.6
	2	92	27.0
	≥3	154	45.4
DIM class ^3^			
	1	114	33.4
	2	110	32.5
	3	116	34.1
ADMP class ^4^			
	1	15	4.6
	2	147	43.2
	3	178	52.3
Mammary quarter			
	Front	222	65.3
	Rear	118	34.7
Treatment protocols			
	Amoxicillin + clavulanate	96	28.2
	Cephalexin + kanamycin	86	25.3
	Cephapirin	66	19.4
	Amoxicillin + clavulanate + tylosin	31	9.12
	Tetracycline + neomycin + bacitracin + sulfadoxine + trimethoprim	28	8.24
	Other treatment protocols ^5^	33	9.71

^1^ NIMI: New intramammary infections; ^2^ Recurrence: defined as more than one (but no more than three) CM cases in the same mammary quarter within the same lactation; ^3^ DIM class: Days in milk classes: class 1: 0–100 DIM; class 2: >100 to 200 DIM; class 3: >200 DIM; ^4^ ADMP class: Average daily milk production classes: class 1: <15 L/day; class 2: 15 up to 30 L/day; class 3: >30 L/day; ^5^ Other treatment protocols: Treatment protocols with <20 observations, comprising: (a) amoxicillin + clavulanate + enrofloxacin (*n* = 14), (b) cloxacillin + ampicillin (*n* = 10), (c) amoxicillin + clavulanate + tetracycline (*n* = 4), (d) cephalexin + kanamycin + penicillin (*n* = 2), (e) tetracycline + neomycin + enrofloxacin (*n* = 1), (f) neomycin + tylosin (*n* = 1), and (g) ciprofloxacin + tylosin (*n* = 1).

**Table 3:** Outcome of different protocols per pathogen group for treating clinical mastitis cases.

Pathogen Group	Treatment Protocol	BC ^1^	TF ^2^	Total ^3^
*Streptococcus* spp.		76	36	112
	Amoxicillin + clavulanate	19	3	22
	Amoxicillin + clavulanate + enrofloxacin	0	1	1
	Amoxicillin + clavulanate + tylosin	10	1	11
	Cephalexin + kanamycin	13	17	30
	Cephalexin + kanamycin + penicillin	1	0	1
	Cephapirin	24	10	34
	Ciprofloxacin + tylosin	0	1	1
	Cloxacillin + ampicillin	7	1	8
	Tetracycline + neomycin + enrofloxacin	0	1	1
	Tetracycline + neomycin + bacitracin + sulfadoxine + trimethoprim	2	1	3
*Staphylococcus* spp.		80	36	116
	Amoxicillin + clavulanate	31	15	46
	Amoxicillin + clavulanate + enrofloxacin	4	2	6
	Amoxicillin + clavulanate + tylosin	2	0	2
	Cephalexin + kanamycin	19	8	27
	Cephalexin + kanamycin + neomycin + tetracycline	2	0	2
	Cephapirin	15	11	26
	Cloxacillin + ampicillin	2	0	2
	Tetracycline + neomycin + bacitracin + sulfadoxine + trimethoprim	5	0	5
Enterobacterales		81	13	94
	Amoxicillin + clavulanate	18	4	22
	Amoxicillin + clavulanate + enrofloxacin	5	1	6
	Amoxicillin + clavulanate + tylosin	15	2	17
	Cephalexin + kanamycin	18	5	23
	Cephalexin + kanamycin + neomycin + tetracycline	2	0	2
	Cephalexin + kanamycin + penicillin	1	0	1
	Cefoperazone	2	0	2
	Cephapirin	2	0	2
	Neomycin + tylosin	1	0	1
	Tetracycline + neomycin + bacitracin + sulfadoxine + trimethoprim	17	1	18
*Enterococcus* spp.		11	7	18
	Amoxicillin + clavulanate	4	2	6
	Amoxicillin + clavulanate + enrofloxacin	1	0	1
	Amoxicillin + clavulanate + tylosin	1	0	1
	Cephalexin + kanamycin	2	4	6
	Cephapirin	1	1	2
	Tetracycline + neomycin + bacitracin + sulfadoxine + trimethoprim	2	0	2
Total		248	92	340

^1^ BC: Bacteriological cure; ^2^ TF: treatment failure; ^3^ Total: total number of CM cases of this pathogen group treated with the respective treatment protocol.

**Table 4:** Group and species level frequency of isolation of mastitis-causing pathogens in 340 clinical mastitis cases.

Species/Groups of Species		*n*	%
Gram-positive		245	72.06
*Staphylococcus* spp.		116	34.12
	*Staphylococcus chromogenes*	63	18.53
	*Staphylococcus sciuri*	15	4.41
	*Staphylococcus aureus*	12	3.53
	*Staphylococcus simulans*	7	2.06
	*Staphylococcus hyicus*	4	1.18
	*Staphylococcus epidermidis*	3	0.88
	*Staphylococcus* spp.	3	0.88
	*Staphylococcus haemolyticus*	2	0.59
	*Staphylococcus saprophyticus*	2	0.59
	*Staphylococcus xylosus*	2	0.59
	*Staphylococcus gallinarum*	1	0.29
	*Staphylococcus hylentus*	1	0.29
	*Staphylococcus succinus*	1	0.29
*Streptococcus* spp.		102	30.00
	*Streptococcus uberis*	48	14.12
	*Streptococcus dysgalactiae*	44	12.94
	*Streptococcus agalactiae*	6	1.76
	*Streptococcus equinus*	2	0.59
	*Streptococcus canis*	1	0.29
	*Streptococcus gallolyticus*	1	0.29
	*Lactococcus lactis*	4	1.18
	*Aerococcus viridans*	4	1.18
	*Lactococcus garvieae*	1	0.29
*Enterococcus* spp.		27	7.94
	*Enterococcus saccharolyticus*	9	2.65
	*Enterococcus faecalis*	5	1.47
	*Enterococcus hirae*	2	0.59
	*Enterococcus casseliflavus*	1	0.29
	*Enterococcus faecium*	1	0.29
Gram-negative		95	27.94
	*Escherichia coli*	41	12.06
	*Klebsiella pneumoniae*	13	3.82
	*Klebsiella oxytoca*	7	2.06
	*Serratia marcescens*	6	1.76
	*Enterobacter cloacae*	5	1.47
	*Pseudomonas aeruginosa*	4	1.18
	*Enterobacter asburiae*	3	0.88
	*Citrobacter freundii*	2	0.59
	*Enterobacter bugandensis*	2	0.59
	*Pantoea agglomerans*	2	0.59
	*Pseudomonas putida*	2	0.59
	*Acinetobacter defluvii*	1	0.29
	*Acinetobacter lwoffii*	1	0.29
	*Aeromonas caviae*	1	0.29
	*Pseudomonas chlororaphis*	1	0.29
**Species/Groups of Species**		***n***	**%**
	*Pseudomonas japonica*	1	0.29
	*Salmonella* spp.	1	0.29
	*Serratia liquefaciens*	1	0.29
	*Serratia* spp.	1	0.29
Total		340	100

**Table 5:** Antimicrobial susceptibility profiles of 340 bacterial isolates from mild and moderate clinical mastitis cases, determined by agar disk diffusion.

	*Staphylococcus* spp. (*n* = 116)		*Streptococcus* spp. (*n* = 102)		Strep-Like Bacteria ^1^ (*n* = 36)		Enterobacterales (*n* = 95)	
Antimicrobials	Resistant	%	Resistant	%	Resistant	%	Resistant	%
Penicillin-novobiocin (10 µg)	7	6	2	2	3	11.1	na ^2^	na
Ampicillin (10 µg)	6	5.2	11	10.8	1	3.7	46	48.4
Amoxicillin + clavulanate (30 µg)	3	2.6	3	2.9	3	11.1	19	20
Oxacillin (30 µg)	17	14.7	22	21.6	na	na	na	na
Cephalexin (30 µg)	na	na	9	8.8	na	na	27	28.4
Ceftiofur (30 µg)	4	3.4	3	2.9	na	na	13	13.7
Kanamycin (30 µg)	3	2.6	na	na	na	na	24	25
Gentamicin (10 µg)	1	0.9	na	na	na	na	5	5.3
Neomycin (30 µg)	4	3.4	na	na	na	na	24	25.3
Enrofloxacin (5 µg)	8	6.9	9	8.8	0	0	17	17.9
Clindamycin (2 µg)	20	17.2	20	19.6	19	70.4	na	na
Tetracycline (1 µg)	16	13.8	32	31.4	6	22.2	19	20

^1^ Strep-like bacteria: encompassing *Enterococcus* spp. *Lactococcus* spp. *Aerococcus* spp.; ^2^ na: Not available information regarding the breakpoint for inhibition zone interpretation in the CLSI manual.

**Table 6:** Results of the generalized linear mixed model for bacteriological cure of clinical mastitis cases (*n* = 275).

Independent Variable		Estimate	SE ^1^	*p*-Value	OR ^2^	95% CI ^3^
Intercept		−0.16	0.99	0.874	0.86	(0.12 to 6.23)
Clinical mastitis severity score						
	(mild/moderate)	−0.01	0.51	0.985	1.01	(0.38 to 2.68)
Parity		0.06	0.11	0.591	1.06	(0.86 to 1.30)
Pathogen						
	(*Enterococcus* spp. vs. others)	−0.52	0.85	0.538	0.59	(0.11 to 3.09)
	(*Staphylococcus* spp. vs. others)	−0.26	0.52	0.615	0.77	(0.28 to 2.15)
	(*Streptococcus* spp. vs. others)	−0.03	0.58	0.956	0.97	(0.31 to 3.05)
Treatment protocols						
	Tetracycline + neomycin + bacitracin + sulfadoxine + trimethoprim	3.20	1.54	0.038 *	24.53	(1.18 to 510.1)
	Amoxicillin + clavulanate	0.74	0.71	0.296	2.10	(0.52 to 8.52)
	Amoxicillin + clavulanate + tylosin	2.62	1.32	0.047 *	13.79	(1.04 to 183.5)
	Cephapirin	0.36	0.73	0.626	1.43	(0.34 to 6.03)
	Other treatment protocols	1.16	0.77	0.131	3.18	(0.70 to 14.40)
Multidrug resistance						
	(yes/no)	1.14	0.62	0.087	3.13	(0.93 to 10.58)
Number of antimicrobial resistances		−0.36	0.26	0.168	0.70	(0.42 to 1.19)

^1^ SE: Standard error; ^2^ OR: odds ratio; ^3^ CI: confidence interval. * Statistically significant at the 95% confidence level.

## Data Availability

The data presented in this study are available on request from the corresponding author. Data are not publicly available due to privacy or ethical restrictions regarding the farms involved in the study.

## Abbreviations

The following abbreviations are used in this manuscript:

AIC	Akaike Information Criterion
AMR	Antimicrobial resistance
AMU	Antimicrobial use
AST	Antimicrobial susceptibility test
BC	Bacteriological cure
CEUA	Ethics Committee on the Use of Animals
CM	Clinical mastitis
D0	Pretreatment (Day 0)
D14	14 ± 3 days
D21	21 ± 3 days
GLMM	Generalized linear mixed model
k	Cohen’s Kappa coefficient

## References

[1] O’Neill J. (2015). Antimicrobials in Agriculture and the Environment: Reducing Unnecessary Use and Waste the Review on Antimicrobial Resistance.

[2] WHO (2000). WHO Global Principles for the Containment of Antimicrobial Resistance in Animals Intended for Food.

[3] WHO (2005). Critically Important Antibacterial Agents for Human Medicine for Risk Management Strategies of Non-Human Use: Report of a WHO Working Group Consultation.

[4] Tang K.L., Caffrey N.P., Nóbrega D.B., Cork S.C., Ronksley P.E., Barkema H.W., Polachek A.J., Ganshorn H., Sharma N., Kellner J.D. (2017). Restricting the Use of Antibiotics in Food-Producing Animals and Its Associations with Antibiotic Resistance in Food-Producing Animals and Human Beings: A Systematic Review and Meta-Analysis. Lancet Planet. Health.

[5] Simjee S., Ippolito G. (2022). European Regulations on Prevention Use of Antimicrobials from January 2022. Rev. Bras. Med. Vet..

[6] Saini V., McClure J.T., Scholl D.T., DeVries T.J., Barkema H.W. (2013). Herd-Level Relationship between Antimicrobial Use and Presence or Absence of Antimicrobial Resistance in Gram-Negative Bovine Mastitis Pathogens on Canadian Dairy Farms. J. Dairy Sci..

[7] Tomazi T., dos Santos M.V. (2020). Antimicrobial Use for Treatment of Clinical Mastitis in Dairy Herds from Brazil and Its Association with Herd-Level Descriptors. Prev. Vet. Med..

[8] de Jong E., McCubbin K.D., Speksnijder D., Dufour S., Middleton J.R., Ruegg P.L., Lam T.J.G.M., Kelton D.F., McDougall S., Godden S.M. (2023). Invited Review: Selective Treatment of Clinical Mastitis in Dairy Cattle. J. Dairy Sci..

[9] Food and Agriculture Organization (FAO), World Organization for Animal Health (OIE), and World Health Organization (WHO). (2003). FAO OIE WHO Joint FAO/OIE/WHO Expert Workshop on Non-Human Antimicrobial Usage and Antimicrobial Resistance: Scientific Assessment.

[10] Lago A., Godden S.M., Bey R., Ruegg P.L., Leslie K. (2011). The Selective Treatment of Clinical Mastitis Based on On-Farm Culture Results: I. Effects on Antibiotic Use, Milk Withholding Time, and Short-Term Clinical and Bacteriological Outcomes. J. Dairy Sci..

[11] Lago A., Godden S.M. (2018). Use of Rapid Culture Systems to Guide Clinical Mastitis Treatment Decisions. Vet. Clin. N. Am.—Food Anim. Pract..

[12] Granja B.M., Fidelis C.E., Garcia B.L.N., dos Santos M.V. (2021). Evaluation of Chromogenic Culture Media for Rapid Identification of Microorganisms Isolated from Cows with Clinical and Subclinical Mastitis. J. Dairy Sci..

[13] Nobrega D.B., Naqvi S.A., Dufour S., Deardon R., Kastelic J.P., De Buck J., Barkema H.W. (2020). Critically Important Antimicrobials Are Generally Not Needed to Treat Nonsevere Clinical Mastitis in Lactating Dairy Cows: Results from a Network Meta-Analysis. J. Dairy Sci..

[14] Hoe F.G.H., Ruegg P.L. (2005). Relationship between Antimicrobial Susceptibility of Clinical Mastitis Pathogens and Treatment Outcome in Cows. J. Am. Vet. Med. Assoc..

[15] Apparao M.D., Ruegg P.L., Lago A., Godden S., Bey R., Leslie K. (2009). Relationship between in Vitro Susceptibility Test Results and Treatment Outcomes for Gram-Positive Mastitis Pathogens Following Treatment with Cephapirin Sodium. J. Dairy Sci..

[16] Apparao D., Oliveira L., Ruegg P.L. (2009). Relationship between Results of in Vitro Susceptibility Tests and Outcomes Following Treatment with Pirlimycin Hydrochloride in Cows with Subclinical Mastitis Associated with Gram-Positive Pathogens. J. Am. Vet. Med. Assoc..

[17] El Garch F., Youala M., Simjee S., Moyaert H., Klee R., Truszkowska B., Rose M., Hocquet D., Valot B., Morrissey I. (2020). Antimicrobial Susceptibility of Nine Udder Pathogens Recovered from Bovine Clinical Mastitis Milk in Europe 2015–2016: VetPath Results. Vet. Microbiol..

[18] Middleton J.R., Fox L.K., Pighetti G., Petersson-Wolfe C., NMC (2017). Laboratory Handbook on Bovine Mastitis.

[19] Pinzón-Sánchez C., Ruegg P.L. (2011). Risk Factors Associated with Short-Term Post-Treatment Outcomes of Clinical Mastitis. J. Dairy Sci..

[20] Freu G., Tomazi T., Monteiro C.P., Barcelos M.M., Alves B.G., Dos Santos M.V. (2020). Internal Teat Sealant Administered at Drying off Reduces Intramammary Infections during the Dry and Early Lactation Periods of Dairy Cows. Animals.

[21] Barcelos M.M., Martins L., Grenfell R.C., Juliano L., Anderson K.L., dos Santos M.V., Gonçalves J.L. (2019). Comparison of Standard and On-Plate Extraction Protocols for Identification of Mastitis-Causing Bacteria by MALDI-TOF MS. Braz. J. Microbiol..

[22] Conesa A., Dieser S., Barberis C., Bonetto C., Lasagno M., Vay C., Odierno L., Porporatto C., Raspanti C. (2020). Differentiation of Non-Aureus Staphylococci Species Isolated from Bovine Mastitis by PCR-RFLP of GroEL and Gap Genes in Comparison to MALDI-TOF Mass Spectrometry. Microb. Pathog..

[23] Freu G., Gioia G., Gross B., Biscarini F., Virkler P., Watters R., Addis M.F., Franklin-Guild R.J., Runyan J., Masroure A.J. (2024). Frequency of Non-Aureus Staphylococci and Mammaliicocci Species Isolated from Quarter Clinical Mastitis: A 6-Year Retrospective Study. J. Dairy Sci..

[24] CLSI (2025). CLSI VET01S ^TM^ Performance Standards for Antimicrobial Disk.

[25] Martins L., Gonçalves J.L., Leite R.F., Tomazi T., Rall V.L.M., Santos M.V. (2021). Association between Antimicrobial Use and Antimicrobial Resistance of Streptococcus Uberis Causing Clinical Mastitis. J. Dairy Sci..

[26] Piaia J.G., Martins E., Battisti R., Ottobeli B.A., Pantoja J.C.d.F., Gressler L.T. (2025). Antimicrobials in the Management of Bovine Mastitis. Ciência Rural.

[27] CLSI (2024). CLSI M100^TM^, Performance Standards for Antimicrobial Susceptibility Testing.

[28] European Committee on Antimicrobial Susceptibility Testing EUCAST (2025). Breakpoint-Tabelle 15.0. https://www.eucast.org.

[29] Humphries R.M., Hindler J.A. (2014). In Vitro Antimicrobial Susceptibility of Aerococcus Urinae. J. Clin. Microbiol..

[30] Kolar Q.K., Goncalves J.L., Erskine R.J., Ruegg P.L. (2024). Comparison of Minimum Inhibitory Concentrations of Selected Antimicrobials for Non-Aureus Staphylococci, Enterococci, Lactococci, and Streptococci Isolated from Milk Samples of Cows with Clinical Mastitis. Antibiotics.

[31] Bates D., Mächler M., Bolker B.M., Walker S.C. (2015). Fitting Linear Mixed-Effects Models Using Lme4. J. Stat. Softw..

[32] McKinney W. (2011). Pandas: A Foundational Python Library for Data Analysis and Statistics. Python High Perform. Sci. Comput..

[33] Seabold S., Perktold J. (2010). Statsmodels: Econometric and Statistical Modeling with Python. Proceedings of the 9th Python in Science Conference.

[34] Magiorakos A.P., Srinivasan A., Carey R.B., Carmeli Y., Falagas M.E., Giske C.G., Harbarth S., Hindler J.F., Kahlmeter G., Olsson-Liljequist B. (2012). Multidrug-Resistant, Extensively Drug-Resistant and Pandrug-Resistant Bacteria: An International Expert Proposal for Interim Standard Definitions for Acquired Resistance. Clin. Microbiol. Infect..

[35] Suojala L., Kaartinen L., Pyörälä S. (2013). Treatment for Bovine Escherichia Coli Mastitis—An Evidence-Based Approach. J. Vet. Pharmacol. Ther..

[36] Oliver S.P., Almeida R.A., Gillespie B.E., Headrick S.J., Dowlen H.H., Johnson D.L., Lamar K.C., Chester S.T., Moseley W.M. (2004). Extended Ceftiofur Therapy for Treatment of Experimentally-Induced Streptococcus Uberis Mastitis in Lactating Dairy Cattle. J. Dairy Sci..

[37] McDougall S., Arthur D.G., Bryan M.A., Vermunt J.J., Weir A.M. (2007). Clinical and Bacteriological Response to Treatment of Clinical Mastitis with One of Three Intramammary Antibiotics. N. Z. Vet. J..

[38] Tomazi T., Sumnicht M., Tomazi A.C.C.H., Silva J.C.C., Bringhenti L., Duarte L.M., Silva M.M.M., Rodrigues M.X., Bicalho R.C. (2021). Negatively Controlled, Randomized Clinical Trial Comparing Different Antimicrobial Interventions for Treatment of Clinical Mastitis Caused by Gram-Positive Pathogens. J. Dairy Sci..

[39] Svennesen L., Skarbye A.P., Farre M., Halasa T., Denwood M., Kirkeby C. (2023). Treatment of Mild to Moderate Clinical Bovine Mastitis Caused by Gram-Positive Bacteria: A Noninferiority Randomized Trial of Local Penicillin Treatment Alone or Combined with Systemic Treatment. J. Dairy Sci..

[40] Ruegg P.L. (2018). Making Antibiotic Treatment Decisions for Clinical Mastitis. Vet. Clin. N. Am.—Food Anim. Pract..

[41] Côté-Gravel J., Malouin F. (2019). Symposium Review: Features of Staphylococcus Aureus Mastitis Pathogenesis That Guide Vaccine Development Strategies. J. Dairy Sci..

[42] Fidelis C.E., Orsi A.M., Freu G., Gonçalves J.L., dos Santos M.V. (2024). Biofilm Formation and Antimicrobial Resistance of Staphylococcus Aureus and Streptococcus Uberis Isolates from Bovine Mastitis. Vet. Sci..

[43] De Buck J., Ha V., Naushad S., Nobrega D.B., Luby C., Middleton J.R., De Vliegher S., Barkema H.W. (2021). Non-Aureus Staphylococci and Bovine Udder Health: Current Understanding and Knowledge Gaps. Front. Vet. Sci..

[44] Fusar Poli S., Locatelli C., Monistero V., Freu G., Cremonesi P., Castiglioni B., Lecchi C., Longheu C.M., Tola S., Guaraglia A. (2025). Staphylococcus Aureus and Methicillin-Resistant Staphylococci and Mammaliicocci in the Bulk Tank Milk of Dairy Cows from a Livestock-Dense Area in Northern Italy. Res. Vet. Sci..

[45] Pillar C.M., Stoneburner A., Shinabarger D.L., Abbeloos E., Goby L. (2014). In Vitro Susceptibility of Bovine Mastitis Pathogens to a Combination of Penicillin and Framycetin: Development of Interpretive Criteria for Testing by Broth Microdilution and Disk Diffusion. J. Dairy Sci..

[46] de Jong A., El Garch F., Simjee S., Moyaert H., Rose M., Youala M., Siegwart E. (2018). Monitoring of Antimicrobial Susceptibility of Udder Pathogens Recovered from Cases of Clinical Mastitis in Dairy Cows across Europe: VetPath Results. Vet. Microbiol..

